# Effects of Pilates exercise on glycated hemoglobin and fasting blood sugar in type 2 diabetes: a systematic review and meta-analysis

**DOI:** 10.7586/jkbns.25.046

**Published:** 2025-08-26

**Authors:** Se-Na Yu, Hee-Jin Moon, Myung-Haeng Hur

**Affiliations:** 1 Department of Nursing, Nowon Eulji University Hospital, Seoul, Korea; 2 Department of Nursing, Uijeongbu Eulji University Hospital, Uijeongbu, Korea; 3 College of Nursing, Eulji University, Uijeongbu, Korea

**Keywords:** Diabetes mellitus, Exercise therapy, Glycated hemoglobin, Systematic review, Meta-analysis

## Abstract

**Purpose:**

This systematic review and meta-analysis aimed to evaluate the effects of Pilates exercise on glycemic control—specifically glycated hemoglobin (HbA1c) and fasting blood sugar (FBS)—in patients with type 2 diabetes.

**Methods:**

Following the PICOS framework, eligible studies included: participants (P) diagnosed with type 2 diabetes; interventions (I) involving Pilates exercise regardless of type; comparisons (C) with standard diabetes care; outcomes (O) of HbA1c and FBS; and study design (S) limited to randomized controlled trials. Literature was searched in PubMed, Embase, CINAHL, Cochrane, CNKI, Wanfang, VIP Information, DBpia, KISS, RISS, and ScienceON up to April 2025. A total of 368 studies were identified, of which eight were included in the systematic review and seven in the meta-analysis.

**Results:**

Pilates exercise significantly improved glycemic outcomes compared to standard care: HbA1c (n = 275, mean difference [MD] = −0.96%; 95% confidence interval [CI]: −1.39 to −0.52; *p* < .001) and FBS (n = 231, MD = −23.98mg/dL; 95% CI: −35.89 to −12.07; *p* < .001).

**Conclusion:**

Pilates exercise may be effective as an intervention for reducing HbA1c and FBS levels in individuals with type 2 diabetes. These findings support its clinical application in diabetes management.

## INTRODUCTION

The global prevalence of diabetes continues to rise. In South Korea, the prevalence among individuals aged 30 and older increased from 13.8% in 2018 to 14.8% in 2022 [1]. According to the International Diabetes Federation, approximately 537 million people were living with diabetes in 2021, and this number is projected to increase to 643 million by 2030 [2]. Diabetes is a chronic condition that is difficult to cure and often associated with serious complications, including cardiovascular diseases and end-stage renal disease. Therefore, the primary therapeutic goal for individuals with diabetes is to maintain normal blood glucose levels [1].

Interventions for diabetes management typically include pharmacological therapy, dietary modifications, and exercise therapy. Because insulin therapy alone cannot fully address the underlying pathophysiology of diabetes, it is essential to combine medication with lifestyle interventions [3]. However, due to low adherence rates, individuals with diabetes must have a comprehensive understanding of self-care practices and actively incorporate them into their daily lives [4,5]. Previous studies have shown that significant improvements in blood glucose control are achieved when exercise interventions are combined with pharmacological and dietary therapies, rather than applied alone. These findings confirm that exercise therapy is a critical and effective component in the comprehensive management of diabetes [6,7].

During the coronavirus disease 2019 pandemic, periods of social restriction resulted in decreased physical activity [8], weight gain, and elevated glycated hemoglobin (HbA1c) levels [9]. Consequently, engaging in physical activity during leisure time has been widely recommended [10]. Both the American Diabetes Association and the Korean Diabetes Association endorse regular aerobic and resistance exercises as effective strategies for maintaining optimal blood glucose levels and overall health [11,12]. When implementing exercise interventions, it is essential to consider patients’ physical and functional capacities [13].

Pilates exercise is a full-body workout that emphasizes posture correction, realignment of body mechanics, and core muscle strengthening [11]. It has been shown to improve mobility, physical fitness, muscle activation, balance, flexibility, as well as reduce the risk of injury [11,14], making it suitable for individuals of all age groups. Since 2000, research has investigated the application of Pilates in diverse populations. In South Korea, Pilates exercise interventions for patients with diabetes have shown benefits in reducing stress and improving blood lipid profiles [15], while minimizing joint strain [16]. Among elderly participants, Pilates exercise enhanced physical function and dynamic balance [17]. Additionally, studies involving overweight or obese women reported significant reductions in glycated HbA1c following moderate-intensity Pilates exercises [18].

Individuals with diabetes are at increased risk for sarcopenia, fractures, and falls compared to the general population [19]. Pilates exercise enhances core stability and physical balance through full-body strength training, demonstrating positive effects on multiple aspects of physical function [14,20]. Current exercise guidelines for individuals with diabetes recommend engaging in resistance and strength training, while also emphasizing the importance of preventing exercise-related injuries [12]. Therefore, incorporating Pilates exercise into diabetes management may contribute to improved glycemic control and enhanced physical performance.

A previous meta-analysis [21] demonstrated that Pilates exercise significantly reduces body weight and body mass index (BMI) in overweight or obese individuals. Another systematic review and meta-analysis [22] confirmed the beneficial effects of Pilates on glycemic control, including reductions in glycated HbA1c, as well as improvements in lipid profiles such as total cholesterol. Although the overall effect size for participants with diabetes was statistically significant in that study, it also reported a high degree of heterogeneity, and the reduction in fasting blood sugar (FBS) was not statistically significant. Given that FBS is still considered a useful secondary indicator of short-term glycemic response [12], it was included in the present study to complement the primary outcome of HbA1c and to provide a more comprehensive assessment of glycemic control in individuals with type 2 diabetes.

Therefore, the present study was designed to evaluate the effects of Pilates exercise specifically in individuals with type 2 diabetes, in order to provide more targeted and reliable evidence for this population. Body weight, BMI, and total cholesterol are commonly used indicators of obesity and general metabolic health [23]. However, HbA1c and FBS are more direct and clinically relevant indicators of glycemic control in patients with diabetes [12]. This study conducted a systematic review and meta-analysis of randomized controlled trials investigating the effects of Pilates exercise interventions specifically in individuals with type 2 diabetes. To ensure clinical relevance and consistency, the analysis was limited to studies that measured glycemic control using HbA1c and FBS as outcome variables.

## METHODS

### 1. Study design

This study is a systematic review and meta-analysis that synthesizes qualitative and quantitative data from randomized controlled trials (RCTs) to evaluate the effects of Pilates exercise in individuals with diabetes.

To ensure transparency in the research process, systematic reviews are recommended to register their protocols in PROSPERO (https://www.crd.york.ac.uk/PROSPERO/) prior to study initiation. Accordingly, the protocol for this study was registered in PROSPERO (CRD42024593836) to secure research transparency.

### 2. Inclusion and exclusion criteria

The clinical question for this systematic review was formulated using the PICOS framework [24]. The study population (P) comprised adults diagnosed with type 2 diabetes mellitus. The intervention (I) was Pilates exercise, applied as a therapeutic approach for diabetes management regardless of the specific form or type. The comparison (C) group received standard or general treatments for diabetes. The primary outcome (O) was glycated HbA1c levels measured following the intervention, and the secondary outcome (O) was FBS levels. The study design (S) was limited to RCTs.

### 3. Search strategy

The search strategy for this study involved identifying peer-reviewed journal articles and dissertations published up to April 2025 through electronic databases, including PubMed, EMBASE, CINAHL, the Cochrane Central Register of Controlled Trials (CENTRAL), China National Knowledge Infrastructure (CNKI), Wanfang, VIP Information, DBpia, Korean Studies Information Service System (KISS), Research Information Sharing Service (RISS), and ScienceON. To enhance search sensitivity, gray literature—such as theses, conference presentations, and news articles—was also manually searched in addition to the database search.

The search strategy applied Medical Subject Headings (MeSH) terms and text words combined with Boolean operators (AND/OR) and truncation searches. For international databases, MeSH terms were used to identify studies targeting patients (P) with type 2 diabetes, using terms such as "DM," "Diabetic," "Diabetes," "Type 2 DM," "Diabetes Mellitus [MeSH]," and "Diabetes Mellitus, Type 2 [MeSH]." The intervention (I) was searched using the term "Pilates exercise." Outcome-related (O) search terms included "Glycated hemoglobin [MeSH]," "HbA1c," "Fasting blood sugar," and "Glucose".

Specifically, the search strategy incorporated terms for targeting patients (P) ((DM) OR (Diabetic) OR (Diabetes) OR (Type 2 DM) OR (Diabetes Mellitus) OR (Diabetes Mellitus, Type 2)), intervention (I) (Pilates), and outcome-related (O) terms ((Glycated hemoglobin) OR (HbA1c) OR (Fasting blood sugar) OR (Glucose)). Database-specific filters were applied to optimize search sensitivity and specificity. For Chinese databases, the search strategy for targeting patients (P) employed the terms "Diabetes" (糖尿病), "Type 2 Diabetes" (2型糖尿病), "Diabetes", "Diabetic", and "Diabetes Mellitus [MeSH]". For the intervention (I), all studies utilizing the term "Pilates" (普拉提) were extracted. For Korean databases without MeSH search functionality, the search was conducted using the search terms “Diabetes” (당뇨) and “Pilates” (필라테스).

### 4. Selection process of studies

This systematic review included RCTs that examined the effects of Pilates exercise on individuals with type 2 diabetes. When duplicate publications—such as dissertations and journal articles—were identified, peer-reviewed journal articles were prioritized.

Studies were excluded if they involved animal experiments or preclinical trials, included participants with type 1 diabetes, did not provide access to the full text, were not published in Korean, English, or Chinese, or were non-experimental studies such as reviews or observational studies. In addition, unpublished dissertations and studies that combined Pilates exercise with other interventions were excluded. Data were collected through both online searches of academic search databases and manual searches, focusing on studies evaluating the effects of Pilates exercise on blood glucose control in patients with type 2 diabetes.

### 5. Quality assessment

The quality of the included studies was assessed using the Risk of Bias 2.0 (RoB 2.0) tool [25], an updated version of the Cochrane Risk of Bias tool. RoB 2.0 evaluates the risk of bias in RCTs based on five domains applicable to study results. These domains include bias arising from the randomization process, bias due to deviations from intended interventions, bias due to missing outcome data, bias in measurement of the outcome, and bias in selection of the reported result [26]. A key feature of RoB 2.0 is the inclusion of signaling questions within each domain. Responses to these questions are categorized as ‘Yes (Y),’ ‘Probably Yes (PY),’ ‘Probably No (PN),’ ‘No (N),’ and ‘No Information (NI).’ Based on these responses, an algorithm determines the level of risk of bias for each domain as low risk, some concerns, or high risk [27].

The overall risk of bias for a study is determined by synthesizing the judgments across all five domains. If all domains are judged as low risk, the study is classified as having a low risk of bias. If at least one domain is judged as some concerns, the study is classified as having some concerns, and if any domain is judged as high risk, the study is classified as having a high risk of bias [25,27].

### 6. Statistical analysis

The selected studies were analyzed using Cochrane Review Manager version 5.4 (The Cochrane Collaboration, London, UK). After analyzing the characteristics of the studies included in the systematic review, data were extracted using Excel for the following items: first author, publication year, country, participants’ age, BMI (kg/m²), sample size, main outcome measures, and authors’ conclusions.

For the meta-analysis, either a fixed effects model or a random effects model was selected depending on the degree of heterogeneity among studies. The fixed effects model assumes that the effect size is the same across studies when the participants and interventions are homogeneous; thus, any observed differences in treatment effects between studies are assumed to be due to sampling error. In contrast, the random effects model assumes that the true average treatment effect follows a normal distribution centered around a mean value, with each study representing a random sample from this population. These random effects model is used when homogeneity cannot be assumed or when heterogeneity is present, and it provides wider confidence intervals than the fixed effects model [28].

In this study, heterogeneity in time, frequency, and duration of interventions among the included studies was assessed, and the random effects model was used accordingly. The effect sizes for continuous or quantitative data were analyzed using means and standard deviations, and the main outcome variables (HbA1c and FBS) were measured in the same way across studies, allowing for analysis using the mean difference (MD) between intervention and control groups [29].

Heterogeneity in meta-analysis can arise from diversity, chance, or bias among the included studies. To assess heterogeneity, forest plots were examined and Higgins’ I^2^ statistic was calculated [28]. An I^2^ value of up to 25% was interpreted as low heterogeneity, up to 50% as moderate heterogeneity, and over 75% as high heterogeneity [28,30]. Publication bias was visually assessed using funnel plots, and it is recommended that publication bias be evaluated only when more than 10 studies are included [31].

### 7. Ethical Considerations

This study is a meta-analysis that did not involve the direct collection of data from human subjects. No personally identifiable information or human-derived materials were collected or recorded. Furthermore, no new data were obtained from participants. As the study exclusively used publicly available, pre-existing data, it was reviewed by the Institutional Review Board and was granted an exemption from ethical review.

## RESULTS

### 1. Study characteristics

A literature search was conducted using domestic and international databases up to April 2025, and gray literature was identified through manual searching. As a result of applying the search strategy and manual search, a total of 368 studies were initially identified. Two researchers independently reviewed the titles and abstracts for the first screening. After excluding 108 duplicate records, 260 studies remained. For the second screening, two researchers independently reviewed the full texts of the remaining studies. Of these, 235 studies were excluded because their study design, participants, or interventions were not relevant to the key research question, leaving 25 studies. Among these, nine studies without available full text and two studies published in languages other than English, Chinese, or Korean were excluded. The remaining 14 studies were then reviewed again by two researchers, focusing on the full text. At this stage, studies were excluded if they had no glucose outcome reported (n = 1), did not include a Pilates intervention (n = 1), used a combined intervention with Pilates plus chocolate (n = 1), compared Pilates with another exercise intervention (n = 2), compared Pilates with dietary education (n = 1), were duplicate studies (n = 2), or were a case report (n = 1). After these exclusions, three additional studies identified through manual search were included, resulting in a total of eight studies selected in the second screening.

In total, eight studies were included in the qualitative analysis. Of these, seven studies, which had the same intervention method and outcome variables, clearly reported the number of participants in the experimental and control groups, and presented clear post-intervention results, were included in the meta-analysis. The literature selection process was independently conducted by one doctoral nursing student and one nursing professor, and there were no disagreements.

**Figure 1.**
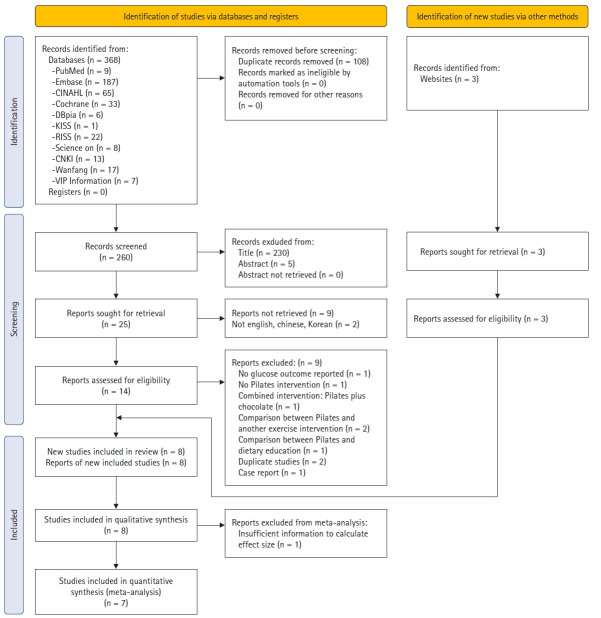
Flow diagram of the study selection process.

To clearly and systematically present the step-by-step literature selection process, the Preferred Reporting Items for Systematic Reviews and Meta-Analyses (PRISMA) 2020 flow diagram, as recommended by the PRISMA, was used (Figure 1, Appendix 1).

The characteristics of the eight studies selected for the effects of Pilates exercise in patients with type 2 diabetes are as follows. Among the included studies, one (12.5%) was published in a domestic journal, whereas seven (87.5%) appeared in international peer-reviewed journals. Although a comprehensive review of all available literature was conducted regardless of publication year, all studies included in the final analysis were published after 2010. With respect to country of origin, three studies (37.5%) were conducted in Iran, two (25.0%) in Brazil, and one study each (12.5%) in Korea, China, and Turkey.

The components of the Pilates exercise interventions were analyzed in terms of session duration, frequency, and total intervention period. Session duration was 45 minutes in one study (12.5%), 60 minutes in five studies (62.5%), and over 75 minutes in two studies (25%). Exercise frequency was twice weekly in one study (12.5%), three times weekly in six studies (75%), and daily in one study (12.5%). The intervention period lasted 8 weeks in four studies (50%), 12 weeks in three studies (37.5%), and 24 weeks in one study (12.5%).

The primary outcome variable assessed in the included studies was glycated HbA1c, while FBS was analyzed as a secondary outcome. HbA1c levels were expressed as percentages, and FBS values were reported in either mmol/L or mg/dL. For consistency, FBS values presented in mmol/L were converted to mg/dL for analysis (Table 1).

### 2. Quality assessment of selected studies

**Figure 2.**
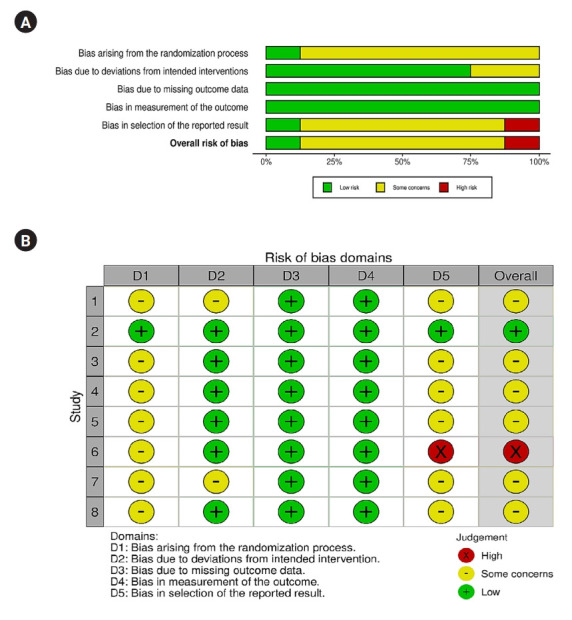
Risk of bias in included studies. (A) Risk of bias graph. (B) Risk of bias of selected studies.

The included studies were critically appraised using the Cochrane Risk of Bias 2.0 (RoB 2.0) tool. The results of the quality assessment were visualized using the ‘robvis’ visualization tool (https://www.riskofbias.info/welcome/robvis-visualizationtool) for systematic review reporting. A summary of the risk of bias findings is presented in Figure 2.

Among the eight studies analyzed, one study (A2, 12.5%) was judged to have an overall low risk of bias, six studies (A1, A3, A4, A5, A7, A8; 75.0%) were rated as having some concerns, and one study (A6, 12.5%) was found to have a high risk of bias. In the quality assessment, the domains most commonly rated as low risk were bias due to missing outcome data and bias in measurement of the outcome. Conversely, the domain most frequently judged as high risk of bias was bias in selection of the reported result. For the randomization process, only one study (A2, 12.5%) was assessed as low risk of bias, while the remaining seven studies (A1, A3, A4, A5, A6, A7, A8; 87.5%) were rated as having some concerns. In terms of deviations from intended interventions, six studies (A2, A3, A4, A5, A6, A8; 75.0%) were assessed as low risk of bias, while two studies (A1, A7; 25.0%) were rated as having some concerns. All studies were judged to have a low risk of bias for missing outcome data and measurement of the outcome. For the selection of the reported result, one study (A2, 12.5%) was rated as low risk of bias, six studies (A1, A3, A4, A5, A7, A8; 75.0%) as having some concerns, and one study (A6, 12.5%) as high risk of bias.

### 3. Effects of Pilates exercise on glycemic control in patients with type 2 diabetes

In this study, eight articles were included in the qualitative analysis. However, one study (A6) did not provide outcome values and was therefore excluded from the quantitative synthesis. Consequently, a meta-analysis examining the effects of Pilates exercise on patients with diabetes was conducted using seven studies for which effect size data were available.

For glycemic control, seven studies (A1, A2, A3, A4, A5, A7, A8) were included in the effect size analysis of glycated HbA1c, while six studies (A1, A3, A4, A5, A7, A8) were included in the effect size analysis of FBS. To evaluate the effect sizes on glycemic control according to exercise intervention characteristics such as session duration, frequency and intervention period, subgroup analyses were conducted.

#### 1) Effects of Pilates exercise on HbA1c in patients with type 2 diabetes

Among the seven studies included in the analysis, Pilates exercise demonstrated a statistically significant reduction in HbA1c levels in the experimental group compared to routine care in the control group, with a MD of −0.96% (n = 275; 95% CI: −1.39 to −0.52; Z = 4.30; *p* < .001). HbA1c was reduced in the Pilates group compared to the control group, indicating a potential improvement in glycemic control. Assessment of study homogeneity revealed high heterogeneity (Higgins I^2^ = 85.7%), so a random-effects model was applied for the analysis of the effect size of Pilates exercise.

Subgroup analyses were conducted based on session duration, categorizing studies into those with 60-minute sessions and those with sessions lasting 75 minutes or more, in accordance with the recommendation of 60 minutes of daily exercise [32]. For session durations of 45~60 minutes, the effect size of Pilates exercise on HbA1c was −0.71% (n = 161; MD = −0.71; 95% CI: −1.13 to −0.28; Z = 3.25; *p* = .001), demonstrating a statistically significant reduction compared to the control group. For session durations of 75~150 minutes, the effect size was −1.39% (n = 114; MD = −1.39; 95% CI: −2.27 to −0.51; Z = 3.09; *p* = .002), also showing a statistically significant difference. However, the between-group difference in effect size according to session duration was not statistically significant (*p* = .170).

Subgroup analysis based on session frequency was conducted in accordance with the guideline recommending exercise at least three times per week [32], categorizing studies into those with fewer than four sessions per week and those with four or more. For session frequencies of 2~3 times per week, the effect size of Pilates exercise on HbA1c was −0.83% (n = 185; MD = −0.83; 95% CI: −1.10 to −0.57; Z = 6.25; *p* < .001), indicating a statistically significant reduction. For a session frequency of seven times per week, the effect size was −1.85% (n = 90; MD = −1.85; 95% CI: −2.14 to −1.56; Z = 12.36; *p* < .001), also demonstrating statistical significance. Moreover, the difference in effect sizes between the subgroups based on session frequency was statistically significant (*p* < .001).

**Figure 3.**
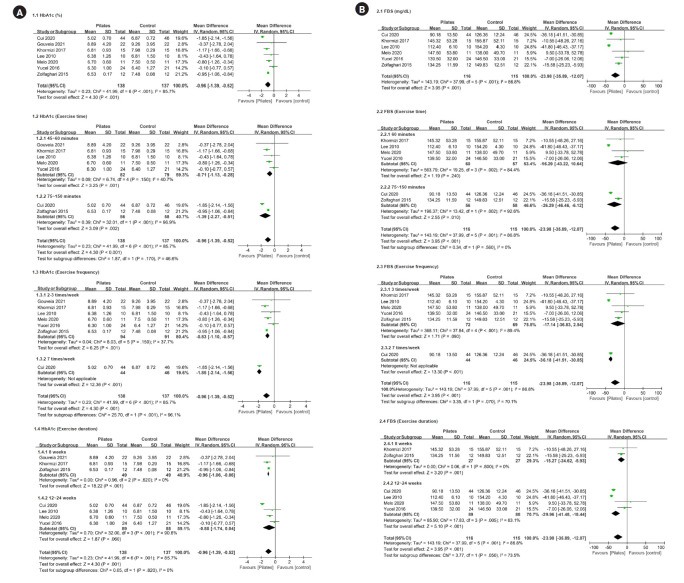
(A) Forest plot of the effect of the Pilates exercise on glycated hemoglobin. (B) Forest plot of the effect of the Pilates exercise on fasting blood sugar. HbA1c = Glycated hemoglobin; SD = Standard deviation; CI= Confidence interval; FBS = Fasting blood sugar.

Subgroup analysis based on intervention period was conducted using a 12-week cutoff, in line with previous studies that examined the effects of 12 weeks of moderate-intensity exercise on glycemic control in patients with diabetes [33]. For an 8-week intervention period, the effect size of Pilates exercise on HbA1c was −0.96% (n = 98; MD = −0.96; 95% CI: −1.06 to −0.86; Z = 18.22; *p* < .001), indicating a statistically significant reduction. For intervention periods of 12~24 weeks, the effect size was −0.85% (n = 177; MD = −0.85; 95% CI: −1.74 to 0.04; Z = 1.87; *p* = .060), which was not statistically significant. The difference in effect sizes between the subgroups based on intervention period was also not statistically significant (*p* = .820) (Figure 3-A).

#### 2) Effects of Pilates exercise on FBS in patients with type 2 diabetes

Of the seven studies included in the analysis, six provided data for the effect size analysis of FBS. The effect size of Pilates exercise on FBS in the experimental group, compared to routine care in the control group, was −23.98 mg/dL (n = 231; MD = −23.98; 95% CI: −35.89 to −12.07; Z = 3.95; *p* < .001), indicating a statistically significant reduction. FBS was reduced in the Pilates group compared to the control group, indicating a potential improvement in glycemic control. Assessment of heterogeneity revealed substantial variability across studies (Higgins’ I^2^ = 86.8%), and thus a random-effects model was employed for the analysis.

Subgroup analyses were conducted based on session duration, categorizing studies into those with 60-minute sessions and those with sessions lasting 75 minutes or more, in accordance with the recommendation of 60 minutes of daily exercise [32]. For session durations of 45~60 minutes, the effect size of Pilates exercise on FBS was −16.29 mg/dL (n = 117; MD = −16.29; 95% CI: −43.22 to 10.64; Z = 1.19; *p* = .240), which was not statistically significant. In contrast, for session durations of 75~150 minutes, the effect size was −26.29 mg/dL (n = 114; MD = −26.29; 95% CI: −46.46 to −6.12; Z = 2.55; *p* = .010), indicating a statistically significant reduction. However, the between-group difference in effect size based on session duration was not statistically significant (*p* = .560).

Subgroup analyses were performed based on session frequency, in accordance with the guideline recommending at least three sessions per week [32], categorizing studies into those with fewer than four sessions per week and those with four or more. For a session frequency of three times per week, the effect size of Pilates exercise on FBS was −17.14 mg/dL (n = 141; MD = −17.14; 95% CI: −36.83 to 2.54; Z = 1.71; *p* = .090), which was not statistically significant. In contrast, for a session frequency of seven times per week, the effect size was −36.18 mg/dL (n = 90; MD = −36.18; 95% CI: −41.51 to −30.85; Z = 13.30; *p* < .001), indicating a statistically significant reduction. However, the difference in effect sizes between subgroups based on session frequency was not statistically significant (*p* = .070).

Subgroup analyses based on intervention period were conducted using a 12-week cutoff, consistent with previous studies examining the effects of 12 weeks of moderate-intensity exercise on glycemic control in patients with diabetes [33]. For an 8-week intervention period, the effect size of Pilates exercise on FBS was −15.27 mg/dL (n = 54; MD = −15.27; 95% CI: −24.62 to −5.93; Z = 3.20; *p* = .001), indicating a statistically significant reduction. For intervention periods of 12~24 weeks, the effect size was −29.96 mg/dL (n = 177; MD = −29.96; 95% CI: −41.48 to −18.44; Z = 5.10; *p* < .001), also demonstrating statistical significance. Moreover, the difference in effect sizes between subgroups based on intervention period was statistically significant (*p* = .050) (Figure 3-B).

### 4. Publication bias

The funnel plot for the seven studies included in this meta-analysis did not exhibit perfect symmetry. According to Higgins et al. [31], assessment of publication bias using funnel plots is recommended only when at least 10 studies are included in the analysis. Therefore, an accurate evaluation of funnel plot asymmetry or the presence of publication bias was not feasible in this study.

## DISCUSSION

This study systematically reviewed the effects of Pilates exercise in patients with type 2 diabetes. After excluding studies that did not meet the inclusion criteria, a total of eight articles were included in the qualitative synthesis, with seven of these subjected to meta-analysis.

The meta-analysis of seven studies found that glycated HbA1c was significantly reduced by 0.96% in the Pilates exercise group compared to the control group. Similarly, FBS was significantly decreased by 23.98 mg/dL in the Pilates group. HbA1c, reflecting the average blood glucose level over the preceding two to three months, is a critical biomarker for diabetes management. It is widely accepted that a 1% change in HbA1c corresponds to an average blood glucose change of 25.2~34.2 mg/dL [34]. This is consistent with the formula used to estimate average glucose (eAG) from HbA1c (eAG [mg/dL] = 28.7 × HbA1c − 46.7), which indicates that a 1% increase in HbA1c corresponds to an approximate rise of 30 mg/dL in average blood glucose levels [35]. Therefore, the reductions in HbA1c and FBS observed in this study can be considered comparable in magnitude.

Previous studies investigating the effects of various exercise interventions in patients with diabetes—including aerobic, resistance, combined exercise, and structured exercise programs—have reported reductions in HbA1c ranging from 0.58% to 0.8% [36-38]. The findings of this meta-analysis suggest that Pilates exercise is comparably effective in lowering HbA1c. Overall, these results support the conclusion that Pilates exercise effectively improves both HbA1c and FBS levels in patients with diabetes.

Pilates is a functional exercise modality known to enhance mobility, physical fitness, and muscle activation [14]. Previous research has suggested that Pilates produces effects comparable to those of aerobic exercise in patients with rheumatoid arthritis [39]. A previous study that applied Pilates exercise to overweight women reported reductions in body weight and BMI, as well as improvements related to insulin resistance [40]. Additionally, the Diabetes Association recommends both aerobic and resistance exercises to achieve optimal glycemic control and promote overall health [11,12]. In light of these guidelines and the findings of the present study, Pilates exercise may be considered a viable and beneficial option for patients with diabetes. Moreover, adults aged 60 and older account for more than half of the age distribution among individuals with diabetes [1]. As this population commonly experiences sarcopenia and functional impairments [41], Pilates may provide additional functional benefits, such as improved balance.

Exercise dose is commonly assessed by parameters such as session duration, session frequency, exercise intensity, or total exercise volume [32]. However, the studies included in this meta-analysis reported only session duration, session frequency, and intervention period. Consequently, subgroup analyses were performed based on session duration (45~60 minutes vs. ≥ 75 minutes), session frequency (2~3 times per week vs. 7 times per week), and intervention period (8 weeks vs. 12~24 weeks).

Subgroup analysis based on session duration revealed that HbA1c was significantly reduced by 0.71% in sessions lasting 45~60 minutes and by 1.39% in sessions of 75 minutes or longer, with no statistically significant difference between the subgroups. Regarding FBS, a significant reduction of 26.29 mg/dL was observed only in the subgroup with session durations of 75 minutes or longer. These findings suggest that longer session durations may exert a more pronounced effect on glycemic control. However, previous studies have reported significant reductions in HbA1c and FBS with combined or aerobic exercise sessions shorter than 30 minutes [42,43], and no significant subgroup differences were detected in the present analysis. Therefore, drawing definitive conclusions regarding the impact of session duration on HbA1c and FBS remains challenging. Notably, as exercise intensity was not specified and relevant data were insufficient, further research is warranted to provide more robust evidence.

In terms of session frequency, HbA1c was reduced by 0.83% with 2~3 sessions per week and by 1.85% with seven sessions per week. FBS was also reduced by 36.18 mg/dL with seven sessions per week, and the reduction in HbA1c between subgroups was statistically significant. However, the feasibility of maintaining such high-frequency exercise in real-world clinical settings must be considered [44], and the available data on Pilates session frequency were limited. Given that current guidelines generally recommend a minimum of three sessions per week [12], the evidence from this subgroup analysis alone is insufficient to support a recommendation of daily Pilates sessions.

When analyzed by intervention period, HbA1c and FBS were reduced by 0.96% and 15.27 mg/dL, respectively, after 8 weeks of Pilates exercise. For interventions lasting 12~24 weeks, HbA1c and FBS were reduced by 0.85% and 29.96 mg/dL, respectively. The difference in mean FBS between the subgroups was statistically significant. While the reduction in HbA1c was statistically significant after 8 weeks of exercise, it was not significant for the 12~24 week intervention period. Given that HbA1c is less influenced by short-term dietary intake than FBS, the findings suggest that the effect of Pilates exercise on glycemic control may be limited over longer intervention periods. However, previous research has shown that 12 weeks of moderate-intensity circuit resistance training significantly reduced HbA1c levels [33]. Considering this and the fact that only four studies in the present meta-analysis examined Pilates interventions of 12~24 weeks, more rigorous studies are needed to draw definitive conclusions about the long-term effects of Pilates exercise. The present results suggest that even a relatively short intervention period of 8 weeks may yield beneficial effects on glycemic control. The absence of significant subgroup differences in HbA1c may be attributed to a plateau effect, wherein the most substantial intervention effects occur within the first 90 days and taper off with extended durations [45].

Previous studies on Tai Chi and yoga in patients with diabetes have reported that long-term exercise interventions may lead to improvements in HbA1c, although such changes are not consistently statistically significant. In contrast, significant reductions in FBS have been observed in some studies [46,47]. These mixed findings may be attributed to small sample sizes and heterogeneity in exercise modalities across studies [48]. Similarly, in the present study, although improvements in HbA1c were observed, they were not consistently statistically significant, whereas FBS showed significant reductions across all intervention periods.

A major strength of this study is that it systematically reviewed the effects of Pilates exercise in individuals with diabetes—a population that requires continuous glycemic management. However, several limitations should be noted. First, the number of randomized controlled trials investigating Pilates exercise in patients with diabetes was relatively small, which limits the overall strength of the evidence. Second, six of the eight included studies involved only female participants, which may limit the generalizability of the findings. Additionally, since BMI is affected by demographic factors, the heterogeneity in participant characteristics may further reduce applicability. Third, some studies lacked detailed descriptions of the specific type or format of Pilates used, and the heterogeneity in participant age may reduce the generalizability of the results. Furthermore, the absence of detailed information regarding exercise intensity impeded accurate estimation of exercise dose. To establish more conclusive evidence on the effects of Pilates exercise on glycemic control, future research should incorporate rigorous and repeated trials with sufficient data on session duration, frequency, intervention period, and intensity.

## CONCLUSION

This systematic review and meta-analysis suggest that Pilates exercise may contribute to improved glycemic control in patients with type 2 diabetes, as reflected by reductions in both glycated HbA1c and FBS levels. These findings suggest that Pilates may serve as a potentially effective non-pharmacological intervention for diabetes management. However, this study has limitations due to the small number of included studies, and further verification through additional research is needed.

## Figures and Tables

**Table 1.** Summary of Pilates Exercise Effects on HbA1c and FBS

Study ID	First author (yr) Country	Age (year) BMI (kg/m^2^)	Intervention (regimen)	Control	Main outcome measures	Pre-intervention M±SD	Post-intervention M±SD	Author's conclusion
A1	Cui (2020) China	I: 69.5; C: 68.0	(I) Diet+ medication, Pilates (90-150 min,7 times/week for 12 weeks, n=44)	(C) Diet+ medication (n = 46)	1) HbA1c (%)	I: 7.79±0.78	I: 5.02±0.70	“Pilates training combined with dietary management effectively controls FPG, PBG and HbA1c and ..."
		I: 22.4; C: 21.9			2) FBS (mg/dL)	C: 7.75±0.76	C: 6.87±0.72	
						I: 155.34±18.36	I: 90.18±13.50	
						C: 154.80±17.64	C: 126.36±12.24	
A2	Gouveia (2021) Brazil	I: 59.1; C: 63.4	(I) Usual diet+ medication, stretching Pilates (classic equipment, 60 min, 2 times/week for 8 weeks, n = 22)	(C) Usual diet+ medication, stretching (n = 22)	1) HbA1c (%)	I: 11.14±2.99	I: 8.89±4.20	"... Pilates protocol reduced the levels of glycated hemoglobin ..."
		I: 27.1; C: 26.8				C: 10.75±3.61	C: 9.26±3.95	
A3	Khormizi (2017) Iran	I: 51.1; C: 51.2	(I) Pilates (60 min, 3 times/week for 8 weeks, n = 15)	(C) Routine care (n = 15)	1) HbA1c (%)	I: 8.75±16.2	I: 6.81±0.93	"Pilates exercise decreases the fasting blood glucose and HbAlc in obese women with type 2 diabetes..."
		I: 30.0; C: 31.7			2) FBS (mg/dL)	C: 8.32±1.94	C: 7.98±0.29	
						I: 161.35±64.62	I: 145.32±53.28	
						C: 153.05±18.48	C: 155.87±52.11	
A4	Lee (2010) Korea	I: 65.3; C: 65.4	(I) Pilates (Mat, 60 min, 3 times/week for 24 weeks, n = 10)	(C) Routine care (n = 10)	1) HbA1c (%)	I: 7.95±1.55	I: 6.38±1.26	"Pilates mat exercise program did not show an effect in improving … showed a positive effect on body composition and FBS, HbA1C.”
		I: 25.7; C: 26.1			2) FBS (mg/dL)	C: 6.78±1.58	C: 6.81±1.50	
						I: 152.10±19.30	I: 112.4±6.10	
						C: 155.80±4.90	C: 154.2±4.30	
A5	Melo (2020) Brazil	I: 65.5; C: 67.5	(I) Diet control +medication, Pilates (60 min, 3 times/week for 12 weeks, n = 11)	(C) Diet control +medication (n = 11)	1) HbA1c (%)	I: 7.80±1.00	I: 6.70±0.60	" Pilates training improves ... chronic and acute glycemic control in older women with type 2 diabetes"
		I: 27.6; C: 30.8			2) FBS (mg/dL)	C: 7.40±0.50	C: 7.50±0.50	
						I: 147.10±63.80	I: 147.50±53.80	
						C: 130.40±46.80	C: 138.00±49.70	
A6	Romuzi (2023) Iran	I: 32.3; C: 31.2	(I) Usual diet +Pilates (60 min, 3 times/week for 8 weeks, n = 11)	(C) Usual diet+ daily activities (n = 11)	-			“The beneficial role of Pilates exercises and the use of royal jelly supplements ... blood sugar control in obese women with type 2 diabetes.”
		I: 30.8; C: 31.3						
A7	Yucel (2016) Turkey	I: 58.5; C: 53.5	(I) Pilates (Mat, 45~70 min, 3 times/week for 12 weeks, n = 24)	(C) Routine care (n = 21)	1) HbA1c (%)	I: 6.70±1.16	I: 6.30±1.00	"Pilates-based mat exercise... improves HbA1c, fasting blood glucose..."
		I: 32.2; C: 30.8			2) FBS (mg/dL)	C: 6.53±1.42	C: 6.40±1.27	
						I: 140±31.00	I: 139.5±32.00	
						C: 131.5±42.00	C: 146.5±33.00	
A8	Zolfaghari (2015) Iran	I: 47.9; C: 48.0	(I) Pilates (75 min, 3 times/week for 8 weeks, n = 12)	(C) Routine care (n = 12)	1) HbA1c (%)	I: 7.45±0.17	I: 6.53±0.17	"Pilates exercises were significant factors in reducing of FBS and HbA1C"
		I: 30.3; C: 31.5			2) FBS (mg/dL)	C: 7.37±0.13	C: 7.48±0.08	
						I: 162.33±12.41	I: 134.25±11.59	
						C: 141.25±14.89	C: 149.83±12.51	

HbA1c = Glycated hemoglobin; FBS = Fasting blood sugar; BMI = Body mass index; M = Mean; SD = Standard deviation; I = Intervention group; C = Control group; FPG = Fasting plasma glucose; PBG = Postprandial blood glucose.

## Data Availability

The data that support the findings of this study are available from the corresponding author upon reasonable request.

## Appendix 1. Studies Included in Systematic Review

A1. Cui P, Wang B, Chang L. The effect of Pilates training combined with diet management on the debilitating state and blood sugar of elderly patients with type 2 diabetes. Chinese Journal of Geriatric Care. 2020;18(5):14-16.

A2. Gouveia SSV, de Morais Gouveia GP, Souza LM, da Costa BC, Iles B, Pinho VA, et al. The effect of pilates on metabolic control and oxidative stress of diabetics type 2–a randomized controlled clinical trial. Journal of Bodywork and Movement Therapies. 2021;27:60-66. https://doi.org/10.1016/j.jbmt.2021.01.004

A3. Khormizi SAT, Azarniveh MS. The effect of Pilates exercise on glycemic control and weight loss in obese women with type2 diabetes. International Scientific Journal of Kinesiology. 2017;10:68-73.

A4. Lee KH, Kim CH, Kim CH. Physical science: the effect of Pilates mat exercise program on the body composition and blood metabolic variable in type 2 elderly diabetic patients. Korea Sports Research. 2010;21(2):85-93.

A5. Melo KCB, de Souza Araújo F, Júnior CCMC, de Andrade KTP, Moreira SR. Pilates method training: functional and blood glucose responses of older women with type 2 diabetes. The Journal of Strength and Conditioning Research. 2020;34(4):1001-1007. https://doi.org/10.1519/jsc.0000000000002704

A6. Romuzi N. The effect of a period of Pilates and the supplementation of Royal Jelly on inflammatory indexes of diabetic obese women. Journal of Sports Physiology and Athletic Conditioning. 2023;7(7):63. https://doi.org/10.52547/jspac.39953.3.7.63

A7. Yucel H, Uysal O. Pilates-based mat exercises and parameters of quality of life in women with type 2 diabetes. Iran Red Crescent Medical Journal. 2016;18(3):e21919. https://doi.org/10.5812/ircmj.21919

A8. Zolfaghari N, Faramarzi M, Afkhami-Ardekani M, Afkhami-Ardekani A, Jam Ashkezari S. The effect of eight weeks Pilates exercise on testosterone and sex hormone biding globulin (SHBG) in women with type 2 diabetes. Iranian Journal of Diabetes and Obesity. 2015;7(2):45-49.
